# Lipid and fatty acid dynamics in mature female albacore tuna (*Thunnus alalunga*) in the western Indian Ocean

**DOI:** 10.1371/journal.pone.0194558

**Published:** 2018-04-02

**Authors:** Zahirah Dhurmeea, Heidi Pethybridge, Chandani Appadoo, Nathalie Bodin

**Affiliations:** 1 Department of Biosciences and Ocean Studies, Faculty of Science, University of Mauritius, Réduit, Mauritius; 2 Research Institute for Sustainable Development (IRD)—Marine Biodiversity, Exploitation & Conservation Unit, Victoria, Mahé, Seychelles; 3 CSIRO Oceans and Atmosphere, Hobart, TAS, Australia; 4 Seychelles Fishing Authority (SFA), Victoria, Mahé, Seychelles; Universitat Politècnica de València, SPAIN

## Abstract

Lipid composition in the reproductive and somatic tissues were investigated for female albacore tuna, *Thunnus alalunga*, in the western Indian Ocean, between latitude 18–21°S and longitude 56–60°E, from January 2014 to March 2015. Highest total lipids (TL) were found in the gonads of spawning-capable females (SCP) (mainly phospholipids, PL, triacylglycerols, TAG and wax esters, WE) and in the liver of females in the late regressing and regenerating ovary phases (mainly TAG, PL and sterols, ST). Muscle TL was low but exhibited high inter-individual variability. Correlations between gonadosomatic and hepatosomatic indices with TL and the lipid classes in albacore gonads and liver describes a pattern of reallocation of energy from the liver to the gonads during SCP. Female albacore were also observed to pursue foraging activities even during this period. Therefore, female albacore can be considered as a capital-income breeder relying mostly on stored lipids before the onset of reproduction and to a lesser extent on energy derived from concurrent feeding during the spawning season. Overall, the three examined tissues had similar general fatty acid profiles with the dominance of 22:6ω3 (docosahexaenoic acid, DHA), 16:0, 18:0 and 18:1ω9. The proportions of fatty acids varied with maturity stage and ovary lobe, with the smaller lobe having significantly higher proportions of essential fatty acids, as well as 16:0 and 18:1n9, compared to the larger one. Our results provide new information on the life-history and energy allocation strategy of albacore which will assist fisheries managers.

## Introduction

Lipids are the main source of metabolic energy in marine fish for swimming, growth and reproduction [1] and lipid levels are known to vary in relation to the reproductive cycle [2,3]. Lipids can be obtained directly from the diet, mobilized from storage tissues to the gonads and from de novo synthesis [4]. Tunas allocate a significant amount of energy into egg production [5] and lipids act as the main source of food for the embryo [4]. In addition, the importance of fatty acids in fish reproduction, especially the omega-6 (ω6) arachidonic acid (AA: 20:4ω6) and those of the omega-3 (ω3) type like eicosapentaenoic acid (EPA: 20:5ω3) and docosahexaenoic acid (DHA: 22:6ω3), in fish reproduction is well established. These fatty acids promote fecundity, the rates of hatching, fertilization, egg viability and survival of fish larvae [6–9]. Thus, a reduction in the amount of energy and essential nutrients could be a constraint for productivity of the fish [10,11] by affecting the viability of its progeny [8]. Changes in the concentrations and proportions of these lipid classes and constituent fatty acids in different tissues throughout oocyte maturation are strongly linked to their functions [12] and the fish reproductive potential [10].

The allocation of energy to resource acquisition, growth, reproduction and survival, is an integral part of life-history. The breeding strategy of a species regulates the partitioning of energy during reproduction [13] with the two main strategies identified as capital and income breeders [14]. Most organisms develop a combination of breeding strategies [15] and adapt the strategy based on their internal and external conditions [14], such as prey (energy and nutrient) availability in their marine environment [16]. Fish species inhabiting temperate waters, where environmental conditions (e.g., day length, temperature and productivity) are highly variable, are expected to store energy seasonally for gonad development [2] prior to reproduce at a later time regardless of food availability (i.e., capital breeder) [16]. Thus, for capital breeders, essential fatty acids required for the early survival of the developing larvae largely originate from the spawner’s lipid reserves [4,17]. In contrast, fish species inhabiting tropical and subtropical regions with relatively constant environmental conditions, are likely to fulfil their energy requirements for reproduction by concurrent feeding (i.e., income breeder) and do not have a mechanism to store energy. These represent the extremes of a continuum. There is no clear separation between the two strategies and species may develop different strategies depending on their environment [15]. The assessment of the cost that reproduction implies and identification of the type of energy allocation strategy is essential since the reproductive potential may depend on endogenous energy reserves rather than on food availability during the spawning season [16].

Migrating fish, like tunas, seem to have a higher and more variable lipid content than the majority of non-migrating fish species [18]. Albacore tuna, *Thunnus alalunga* (Bonnaterre 1788), is a pelagic species occurring worldwide throughout temperate waters, sub-tropical and tropical zones [19]. In the western Indian Ocean, large and mature individuals were found to occur mostly north of 30°S while small and immature ones occurred further south [20,21]. It has also recently been shown that albacore spawn between 10°S and 30°S, mainly to the east of Madagascar, from October to January. Albacore have an asynchronous oocyte development and indeterminate fecundity [21,22]. The fork length (*L*_F_) at which 50% of the population is mature was estimated at 85.3 ± 0.7 cm and the minimum size-at-maturity was 83 cm *L*_F_ [21]. Batch fecundity ranged between 0.26 and 2.09 million eggs and the relative batch fecundity was estimated at 53.4 ± 23.2 oocytes g^-1^ of somatic-gutted body weight. Currently, information on the lipid dynamics involved in reproduction is available only for tropical tunas such as yellowfin (*T*. *albacares*; [3] and skipjack (*Katsuwonus pelamis*; [23]) and other temperate tunas (e.g., in Atlantic bluefin tuna, *T*. *thynnus thynnus*; [2]), as well as various farmed species [24]. To date, most studies that have reported the chemical composition of albacore tissues have focused on the quantification of polyunsaturated fatty acid (PUFA) content in the context of human nutrition [25,26]. More recently, some studies have investigated the spatial variation of fatty acids in muscle tissue of albacore in the southwest Pacific to explore trophodynamics [27] and variations with the environment [28]. Bioenergetic models have shown that albacore populations allocate 43% of consumed energy to active respiration with only 6% to growth and reproduction, reflecting a “periodic” life strategy with late maturation, slow growth, low mortality, and moderate to high reproductive output [29]. Currently, no investigation on the related energetics of albacore tuna that includes the importance of lipids in gonad development, maternal investment and determination of repro-somatic energy allocation patterns has been undertaken. The description of the role of lipid classes and fatty acids in mature albacore could provide better understanding of the reproductive potential of the species in the region [10]. Such studies may also provide baseline information for future research including those that are focused on the potential impacts of climate change on the food web, bioenergetics, behaviour and eventually the reproductive potential of the studied fish.

The present study aims first to investigate the energy allocation strategy of albacore tuna from the western Indian Ocean through the analysis of total lipid content (TL) and lipid classes from somatic and reproductive tissues during and after the spawning season, and by describing their relationships with condition indices. In view of the importance of fatty acids during reproduction, we also describe their variation in the different tissues. Additionally, given the asymmetry in the size of gonads in albacore [22,30–32], the difference in fatty acid composition between the small- and large-sized ovary lobe is also investigated.

## Material and methods

### Sample collection

**Fig 1:**
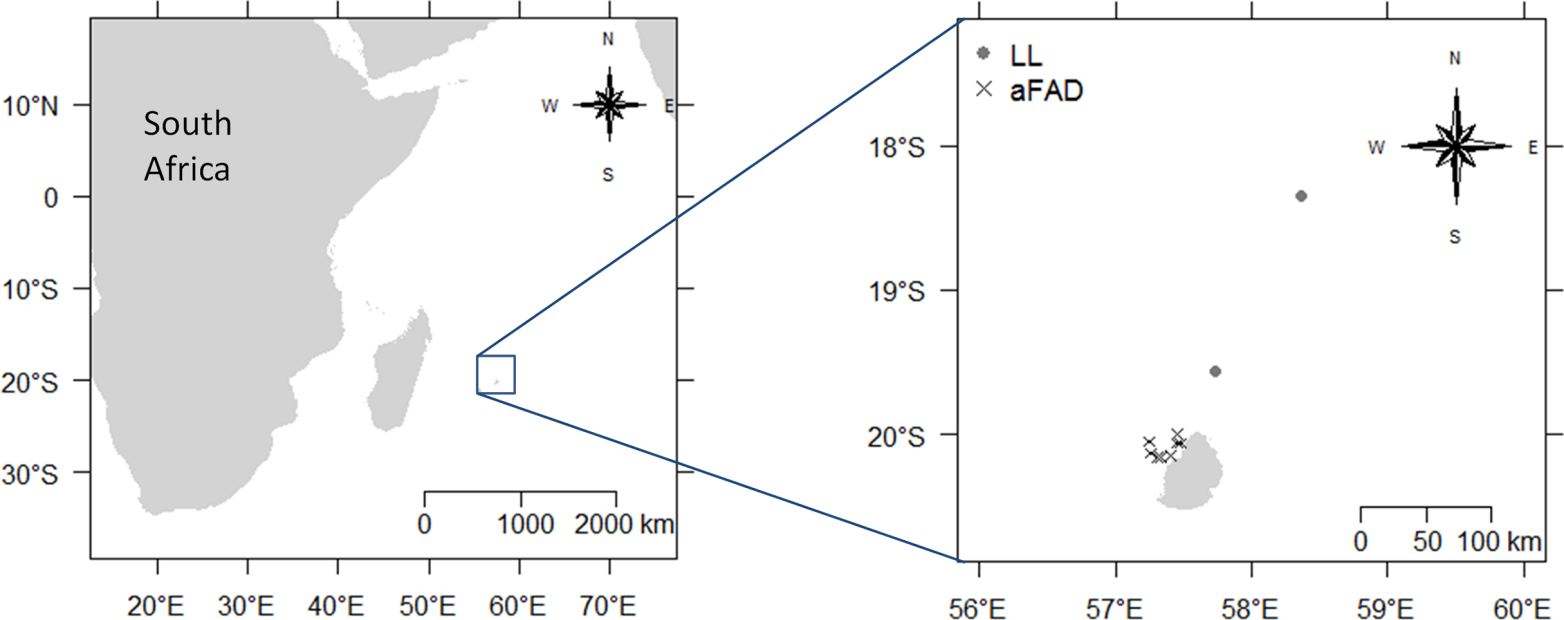
Map of the western Indian Ocean showing the fishing positions of sampled albacore tuna. Grey circles: local longliner (LL), black cross: artisanal coastal fishermen operating around anchored fish aggregating devices (aFADs)].

Albacore caught from January 2014 to March 2015 were sampled in the Exclusive Economic Zone (EEZ) of Mauritius in the western Indian Ocean, between latitude 18–21°S and longitude 56–60°E (Fig 1). Fish were caught either by local longliners or artisanal fishermen fishing around anchored fish aggregating devices (aFADs) set around the coast of Mauritius island. Fish caught by the longliners were sampled at the local processing plants while albacore caught by coastal fishermen on the same day were sampled directly at landing sites. The following measurements were taken from the fish caught around aFADs and by local longliners: *L*_F_, somatic fish weight (*W*_S_) corresponding to the total fish weight minus the total viscera weight, total gonad weight (*W*_G_), trimmed of mesenteric tissue, and liver weight (*W*_L_). For the females, a 4–5 cm cross-section from the middle portion of the larger ovary lobe was collected for consistency and preserved in neutral 4% buffered formaldehyde for histological analysis to assess the maturity stage. In parallel, tissue samples of around 2 g were collected from the liver, the larger ovary lobe and the front dorsal white muscle, and stored frozen at -80°C for lipid and fatty acid analyses. Among the females sampled, 20 individuals in the spawning-capable phase (SCP), first identified macroscopically as described by Brown-Peterson et al. [33], were randomly selected for collection of an additional 2 g-samples from their smaller gonad lobeto compare lipid and fatty acid profiles between the small- and large-sized ovary lobes. Three condition indices were calculated; the gonadosomatic index (*I*_G_), hepatosomatic index (*I*_H_) and Fulton's condition factor (*F*_K_):

$$I_{G}=\frac{W_{G}}{W_{S}}\times 100\;I_{H}=\frac{W_{L}}{W_{S}}\times 100\;F_{K}=\frac{W_{S}}{L_{F}{}^{3}}\times 100$$

All fish sampled under the study were caught by professional fishermen, licensed by the Ministry of Ocean Economy, Marine Resources, Fisheries and Shipping in Mauritius. Fish were already dead by the time of sampling and no ethical approval was required. All institutional, national and international guidelines were also followed during the research work.

### Histological classification

The preserved ovary sections were dehydrated in alcohol solutions, cleared with xylene and embedded in paraffin [21]. Cross-sections of 6 μm were cut from the paraffin blocks, stained with haematoxylin and eosin, and viewed under a light microscope. For each ovary, the presence of the most advanced oocyte stage, signs of previous spawning (maturity markers, such as residual hydrated oocytes and yellowbodies [34]), postovulatory follicles and atresia stage were identified, as described by Dhurmeea et al. [21], and were grouped into the following phases: the immature phase containing the most developed oocytes in the primary growth stage (chromatin nucleolus and perinuclear oocytes), the developing phase characterized by the occurrence of cortical alveoli, vitellogenic 1 and vitellogenic 2 oocyte stages, and the SCP which included the presence of oocytes in the vitellogenic 3, migratory nucleus or hydrated stage and where <50% of the yolked oocytes were atretic (atretic stage 1).

Females in the early regressing phase (RGN) were characterised by ovaries with ≥50% yolked oocytes in alpha atresia (atretic stage 2) while those in late regressing had 100% of yolked oocytes in alpha atresia (atretic stage 3) or only in beta atresia (atretic stage 4). Regenerating females had ovaries containing no viable or atretic yolked oocytes but where maturity markers were present. The ovary phase of the selected females for assessment of the small and large-ovary lobes were also confirmed through histology.

### Lipid and fatty acid analysis

Lipids were extracted from the albacore tissues (gonad: 0.23–1.29 g, liver: 0.27–0.75 g and muscle: 0.62–2.18 g) according to the Bligh and Dyer [35] method. A solution of dichloromethane:methanol:water (10:20:7.5 ml) was added to the tissue in a separatory funnel, shaken at intervals and left overnight. It was then separated into two phases through addition of 10 ml of dichloromethane and saline water (9 g sodium chloride L^-1^). The extract was then collected from the lower dichloromethane phase using a rotary evaporator and transferred into pre-weighed glass vials. Excess solvent was evaporated under nitrogen gas until a constant weight was reached. The final TL weight was calculated by extracting the corresponding vial weight and was expressed in μg mg^-1^ *ww*.

Lipid classes were analyzed according to Pethybridge et al. [36]. Samples were developed in a solvent system of hexane:diethyl ether:acetic acid (70:10:0.1 ml), and run along with standard solutions using an Iatroscan Mark V TH10 thin layer chromatograph (TLC) coupled with a flame ionisation detector (FID). Results of phospholipids (PL), triacylglycerol (TAG), sterols (ST), wax esters (WE) and free fatty acids (FFA) were reported as μg mg^-1^ *ww*.

Fatty acids were identified and quantified following the method by Parrish et al. [37]. The fatty acid methyl esters (FAME) were liberated from the lipid backbone by transmethylation where aliquot samples of TL were heated at 100°C for 2 hours in a mixture of methanol:concentrated hydrochloric acid:dichloromethane (10:1:1). After cooling, 1 ml of water was added to the samples before extraction in hexane:dichloromethane (4:1) solution three times. The mixture was vortexed and centrifuged following each extraction to separate the layers. The upper layer containing the FAME was transferred into vials and evaporated to dryness under nitrogen gas. Gas chromatographic analyses were achieved using an Agilent Technologies 6890N GC (Palo Alto, California, USA) fitted with an HP-5 cross-linked methyl silicone fused silica capillary column (50 x 0.32 mm i.d.), an FID, a splitless injector and an Agilent Technologies 7683 Series auto-sampler and injector. For peak verification, further analyses were made through gas chromatography-mass spectrometry (GC-MS) on a Finnigan Thermoquest system fitted with an on-column injector and using Thermoquest Xcalibur software (Austin, Texas, USA). Fatty acid results were obtained as area of peaks which were then expressed as a proportion of the total identified compounds. Finally, out of the 65 fatty acids identified, 22 of them accounting each for >0.8% in the samples were considered for statistical analysis.

### Data analysis

One-way analysis of variance (ANOVA) was used to study the variability of the log-transformed condition indices (*I*_G_, *I*_H_ and *F*_K_), TL and lipid classes in the somatic and reproductive tissues between ovary phases. The relationships between tissue lipid results (TL and lipid classes) and morphometric condition indices (*F*_K_, *I*_G_ and *I*_H_) were investigated using Pearson's correlation. TL data were ln + 1 transformed when required to achieve normality of the residuals and homogeneity of the variance.

Arc-sinus root squared transformation was applied to the % fatty acid data to improve normality of residuals and homoscedasticity [38] prior to using ANOVA to test for significant differences between ovary phases. Ratios of fatty acids (Σω3/Σω6, DHA/EPA, DHA/AA, EPA/AA) did not require transformation. Post-hoc Tukey test was performed when significant differences were obtained.

The difference in fatty acid profiles between the large- and small-sized ovary lobes of albacore tuna in the SCP was investigated using PRIMER v6 software [39] including principal component analysis (PCA), analysis of similarities (ANOSIM) based on Euclidean distance matrix, and similarity of percentages analysis (SIMPER). The main fatty acids leading to the dissimilarity were then analysed using paired t-tests after data transformation. Maps, t-tests, ANOVAs and regressions were performed using R version 3.2.2 [40]. Maps were built using the package 'rgdal' [41] and data from http://www.diva-gis.org/Data.

## Results

### Variations in condition indices and tissue lipid class composition during albacore spawning

A total of 47 female albacore tuna ranging from 89 to 107 cm *L*_F_ sampled from the EEZ of Mauritius were used for lipid and fatty acid analysis. Due to their low gonad TL (<15 μg mg^-1^ *ww*), females in late regressing and regenerating phases were grouped together into the other post-spawning phase (OPS). All females were mature and included 66% SCP (with vitellogenic 3 and migratory nucleus as most advanced group of oocytes), 17% RGN and 17% OPS (Table 1). The sampled females had *I*_G_ varying from 0.34 to 2.57, with significantly higher values being observed for SCP and RGN compared to OPS (*F*_(2,44)_ = 62.3, *P* < 0.0001). *I*_H_ and *F*_K_ varied from 0.43 to 1.14 and 1.69 to 2.70, respectively, and did not show significant variations with ovary phase (*I*_H_: *F*_(2,43)_ = 1.71, *P* = 0.19; *F*_K_: *F*_(2,44)_ = 3.0, *P* = 0.06).

**Table 1:** Mean (± SD) fork length (*L*_F_, cm), gonadosomatic index (*I*_G_), hepatosomatic index (*I*_H_) and Fulton's condition factor (*F*_K_) for albacore tuna with ovaries in different reproductive phases. SCP = spawning-capable, RGN = early regressing (ovaries with ≥50% yolked oocytes in alpha atresia), OPS = other post-spawning (ovaries with 100% yolked oocytes in atresia and regenerating ovaries), *n =* number of fish.

Reproductive phase	*n*	*L*_F_	*I*_G_	*I*_H_	*F*_K_
SCP	31	100.2±4.5	1.6±0.4	0.7±0.1	2.1±0.2
RGN	8	101.5±5.5	1.4±0.7	0.7±0.1	2.0±0.2
OPS	8	98.1±3.6	0.4±0.1	0.7±0.2	2.0±0.1

**Fig 2:**
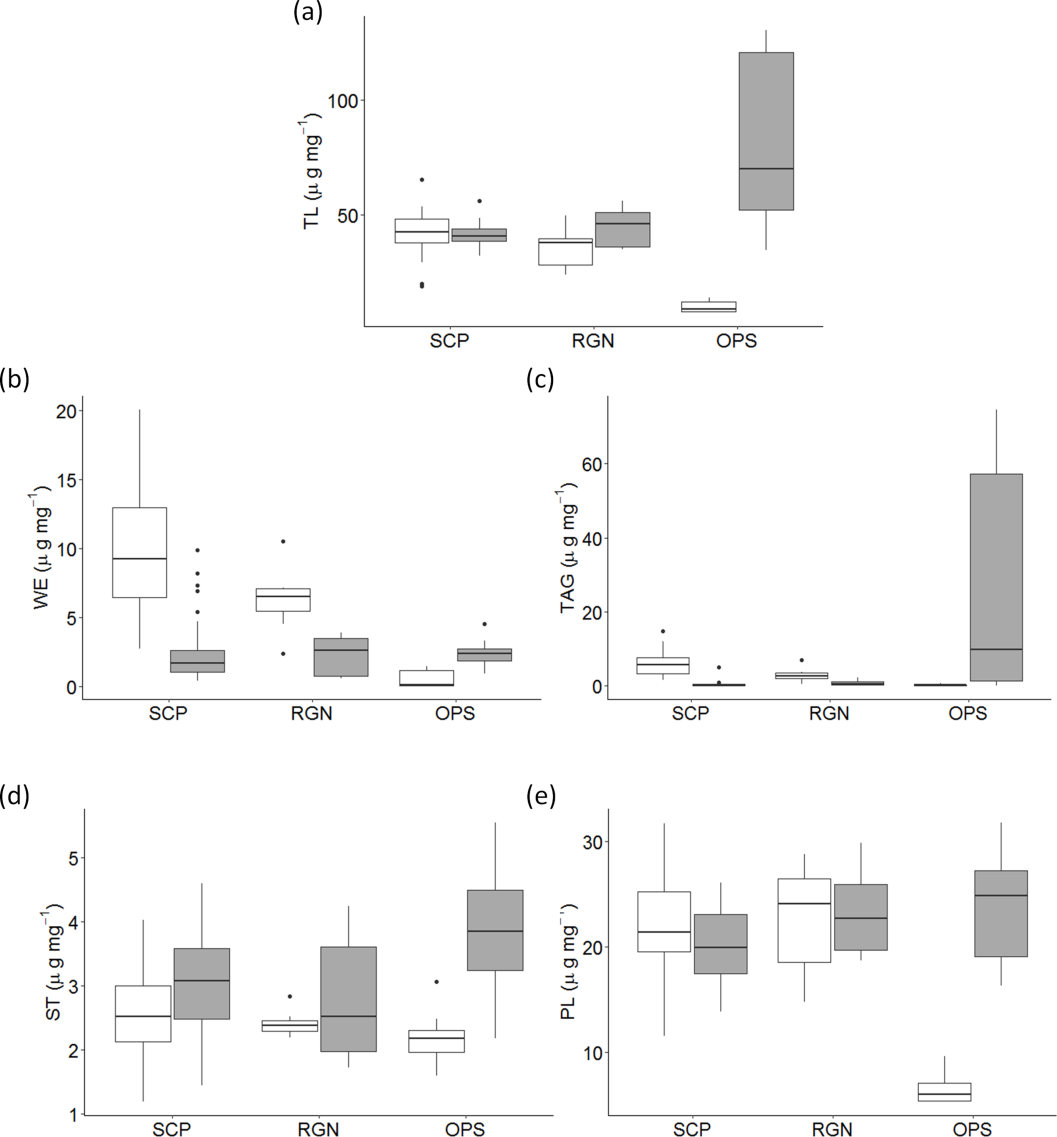
**Boxplots showing variations in (a) total lipid content (TL) levels (**μ**g mg**^**-1**^ **wet weight) and (b)-(e) lipid classes (**μ**g mg**^**-1**^ **wet weight) in gonads (white) and liver (grey) of female albacore tuna with ovary phase**. SCP: spawning-capable, RGN: regressing, OPS: other post-spawning, WE: wax esters, TAG: triacylglycerols, ST: sterols, PL: phospholipids.

The highest concentration of TL was observed in the liver of OPS females (80.3±38.9 μg mg^-1^) which was almost double that observed in liver of SCP (41.5±4.92 μg mg^-1^) and RGN (43.5±8.80 μg mg^-1^) (*F*_(2,44)_ = 16.8, *P* < 0.0001; Fig 2). In the gonads, significantly higher concentrations of TL were observed in the SCP females (43.0±8.59 μg mg^-1^) while OPS gonads had the lowest TL (10.2±2.62 μg mg^-1^) (*F*_(2,44)_ = 40.3, *P* < 0.0001). TL in muscle was very low (around 8 μg mg^-1^) compared to other tissues and was characterised by high inter-individual variance, and did not exhibit any variation with ovary phase (*F*_(2,42)_ = 0.07, *P* = 0.93).

Lipid class composition in the gonads varied depending on the ovary phase although the proportion of PL was predominant contributing to 40–75% of TL compared to the three other classes (around 1–35% of TL) (Fig 2). As observed for TL, PL, WE and TAG showed a significant progressive decrease from SCP to RGN and OPS (PL: *F*_(2,43)_ = 39.4, *P* < 0.0001, WE: *F*_(2,43)_ = 18.0, *P* < 0.0001, TAG: *F*_(2,43)_ = 14.2, *P* < 0.0001). The mean concentrations of PL, WE and TAG were 21.5±4.04, 10.8±4.30 and 6.52±3.15 μg mg^-1^, respectively in the SCP, and decreased to 6.50±1.48, 0.53±0.62 and 0.24±0.24 μg mg^-1^, respectively in OPS. However, the concentration of ST in the ovaries did not vary significantly and remained at around 2 μg mg^-1^ (*F*_(2,43)_ = 1.41, *P* = 0.26).

Unlike the gonads, a significant depletion of TAG in the liver was noted in the SCP where the mean concentration was 0.44±0.89 μg mg^-1^ while higher but variable levels were observed in the liver of OPS females (27.3±32.4 μg mg^-1^) (F_(2,43)_ = 13.8, *P* < 0.0001). ST and PL showed a weak but significant accumulation in the OPS individuals (ST: F_(2,43)_ = 3.59, *P* < 0.05, PL: F_(2,43)_ = 4.12, *P* < 0.05). ST and PL were 3.02±0.76 and 19.9±3.46 μg mg^-1^, respectively in SCP and increased up to 3.87±1.16 and 23.7±5.49 μg mg^-1^ respectively, in OPS.

Finally, none of the lipid classes showed significant differences among the ovary phases in albacore muscle but rather exhibited high individual variability. The main lipid classes in muscle were PL (around 65%) and TAG (around 20%). PL varied from 2.77–6.59 μg mg^-1^ in SCP and 3.17–5.47 μg mg^-1^ in OPS while TAG reached values as high as 14.3 μg mg^-1^ in SCP and 4.52 μg mg^-1^ in OPS. Relative proportions of FFA were low in both muscle and gonad tissues (<10%) but were relatively high in the liver (around 35% in average).

Results of correlation analyses between condition indices and lipid composition of albacore gonads, liver and muscle tissues are summarized in Table 2. *I*_G_ was positively correlated with TL, PL, WE and TAG in the gonads. Likewise, *I*_H_ also showed weak but positive correlations with TL, PL and ST in the gonads. In the liver, decreasing levels of TL, TAG and ST were observed with *I*_G_. In white muscle, the variations in the lipid classes were more apparent with *I*_G_ revealing positive correlations with TL and TAG. A positive correlation was observed between *I*_H_ and *I*_G_ (r = 0.48, *P* < 0.001).

**Table 2:** Summary of correlation analyses between condition indices (*I*_G_, *I*_H_, *F*_K_) with total lipid content (TL) and lipid classes (μg mg^-1^ wet weight) of gonads, liver and muscle of female albacore tuna collected in the waters of Mauritius. WE: wax esters, TAG: triacylglycerols, ST: sterols, PL: phospholipids.

Tissue	Lipid/ lipid class	*I*_G_	*I*_H_	*F*_K_
**Gonads**	TL	0.71****	0.31*	0.30*
	PL	0.79****	0.35*	**0.19**
	TAG	0.65****	**0.25**	0.34*
	ST	0.21	0.32*	-0.07
	WE	0.66****	0.22	0.33*
**Liver**	TL	-0.45**	0.04	-0.39**
	PL	-0.16	-0.08	-0.30*
	TAG	-0.52***	-0.01	-0.35*
	ST	-0.41**	-0.11	-0.01
	WE	-0.01	-0.18	-0.18
**Muscle**	TL	0.40**	0.19	-0.10
	PL	0.06	0.00	0.03
	TAG	0.43**	0.24	-0.04
	ST	0.12	-0.02	0.11
	WE	-0.10	-0.04	0.03

Significant rho values are in bold.

*P < 0.05

**P < 0.01

***P < 0.001

****P < 0.0001.

Similar to *I*_G_, *F*_K_ was positively correlated with TL, WE and TAG in the gonads. On the other hand, in the liver tissue, TL, PL and TAG were negatively correlated with *F*_K_.

### Variations in tissue fatty acid composition during albacore spawning

Fatty acid profiles in the ovary, liver and muscle of female albacore varied from SCP to OPS (Table 3). The proportions of total saturated fatty acids (ΣSFA) in gonads were lower in SCP and RGN compared to OPS (*F*_(2,43)_ = 22.6, *P* < 0.0001) which is mainly due to variations in 18:0 and 17:0. 16:0 composed the majority of SFAs but remained constant around 20% along the different ovarian phases. Total monounsaturated fatty acids (ΣMUFA) were significantly higher in SCP and RGN gonads (*F*_(2,43)_ = 12.8, *P* < 0.0001), mainly as a result of increased proportions of 18:1ω9. With regards to the total polyunsaturated fatty acids (ΣPUFA), no significant variation was observed throughout ovarian phases (*F*_(2,43)_ = 0.59, *P >* 0.05). ΣPUFA accounted for around 40% of the total fatty acids in gonads with DHA constituting more than half of it, especially during SCP and RGN where their proportions were significantly higher (>20% higher) than in OPS. Similarly, EPA proportions in SCP and RGN gonads were significantly higher (around 30% higher) than in OPS gonads. On the other hand, the proportion of arachidonic acid (AA: 20:4ω6) was lowest in SCP gonads and showed an increasing trend through RGN to OPS. Both EPA and DHA mainly drove the variations of %Σω3.% Σω3 was significantly higher in SCP and RGN gonads compared to OPS, which instead was composed of higher proportions of Σω6. DHA/EPA ratio remained constant around 5 in the gonads throughout the three different ovarian phases. The ratios of DHA/AA and EPA/AA in the ovaries were higher for the SCP females compared to the other ovarian phases.

**Table 3:** Mean values (± SD) of fatty acids (as % of total fatty acids) in gonads, liver and muscle of female albacore tuna collected in the waters of Mauritius. Females are classified in different ovary phases: SCP: spawning-capable, RGN: early regressing (ovaries with ≥50% yolked oocytes in alpha atresia), OPS: other post-spawning (ovaries with 100% yolked oocytes in atresia and regenerating ovaries). PL: phospholipids; TAG: triacylglycerols; ST: sterols; WE: wax esters; FFA: free fatty acids; ΣSFA: total saturated fatty acids, ΣMUFA: total monounsaturated fatty acids, ΣPUFA: total polyunsaturated fatty acids, DHA: docosahexaenoic acid, EPA: eicosapentaenoic acid, Σω3: total omega-3 fatty acids, Σω6: total omega-6 fatty acids. *n =* number of samples per tissue.

Tissue	Gonads			Liver			Muscle		
Ovary Phase	SCP	RGN	OPS	SCP	RGN	OPS	SCP	RGN	OPS
*n*	31	7	8	30	8	8	30	7	7
14:0	0.7±0.1	0.7±0.1	0.7±0.1	0.4±0.1	0.4±0.1	0.4±0.0	0.5±0.3	0.5±0.5	0.7±0.5
15:0	0.6±0.0	0.6±0.0	0.6±0.0	0.6±0.1^R^^P^	0.6±0.1^S^^P^	0.5±0.1^S^^R^	0.4±0.2	0.4±0.2	0.4±0.2
16:0	19.9±1.4	20.3±0.7	19.3±0.9	23.7±1.6^P^	24.0±1.0^P^	20.7±2.8^S^^R^	21.2±1.7	21.9±1.2	21.4±0.9
17:0	1.0±0.1^P^	1.0±0.0^P^	1.2±0.1^S^^R^	1.4±0.1^P^	1.5±0.1	1.6±0.2^S^	0.8±0.2^P^	0.9±0.2^P^	1.1±0.3^S^^R^
18:0	6.6±0.7^P^	6.9±0.4^P^	11.9±2.0^S^^R^	11.5±1.2	11.2±1.7	10.7±1.7	8.0±1.2^P^	7.6±0.9^P^	8.7±1.0^S^^R^
Σ**SFA**	**29.1±2.1**^P^	**29.9±1.0**^P^	**35.0±2.9**^S^^R^	**38.6±2.1**^P^	**38.7±1.7**^P^	**34.7±4.4**^S^^R^	**31.6±2.4**^P^	**32.0±1.9**	**33.1±1.8**^S^
16:1ω5	0.4±0.1^P^	0.5±0.1^P^	0.1±0.1^S^^R^	0.1±0.0	0.1±0.0	0.1±0.0	0.1±0.0	0.1±0.0	0.1±0.0
16:1ω7	3.4±0.3^P^	3.3±0.3^P^	1.5±0.4^S^^R^	1.3±0.2	1.5±0.3	1.4±0.4	1.7±1.1	1.7±1.4	1.6±1.1
16:1ω9	0.8±0.1^P^	0.8±0.2^P^	0.3±0.2^S^^R^	0.2±0.0^P^	0.2±0.0	0.3±0.1^S^	0.1±0.1	0.1±0.1	0.1±0.1
18:1ω5	0.4±0.2^P^	0.3±0.2^P^	0.1±0.0^S^^R^	0.1±0.0	0.1±0.0	0.1±0.0	0.1±0.0	0.1±0.0	0.1±0.0
18:1ω7	2.6±0.3^R^	2.3±0.2^S^	2.6±0.2	1.9±0.3^P^	2.1±0.3^P^	2.6±0.6^S^^R^	2.1±0.5	2.0±0.5	1.8±0.5
18:1ω9	12.5±1.5^P^	11.7±0.8^P^	8.4±0.3^S^^R^	8.8±1.1^P^	9.6±1.1	10.9±4.5^S^	11.0±4.0	9.8±3.9	7.8±2.2
20:1ω9	0.8±0.2^P^	0.9±0.2	1.0±0.2^S^	0.8±0.2^R^^P^	1.0±0.3^S^^P^	1.9±0.6^S^^R^	1.2±0.6	1.2±0.8	1.5±0.7
20:1ω11	0.3±0.1	0.3±0.1	0.2±0.2	0.2±0.1^R^^P^	0.3±0.1^S^^P^	0.6±0.2^S^^R^	0.3±0.1^P^	0.3±0.2	0.5±0.2^S^
24:1ω9	0.7±0.2^P^	1.0±0.4^P^	3.6±0.9^S^^R^	1.5±0.2^P^	1.6±0.3^P^	1.0±0.4^S^^R^	1.6±0.6	1.6±0.5	1.4±0.4
24:1ω11	0.1±0.1^P^	0.1±0.0^P^	0.3±0.1^S^^R^	0.1±0.0^R^^P^	0.1±0.0^S^	0.1±0.0^S^	0.1±0.2	0.1±0.0	0.1±0.0
Σ**MUFA**	**23.7±2.1**^P^	**22.4±1.0**^P^	**20.2±0.7**^S^^R^	**16.4±1.7**^R^^P^	**18.4±2.0**^S^	**21.0±5.8**^S^	**19.8±6.3**	**18.5±6.9**	**16.4±4.5**
18:2ω6	0.9±0.1^R^^P^	0.8±0.1^S^^P^	0.6±0.1^S^^R^	0.6±0.1^P^	0.6±0.1	0.7±0.1^S^	0.6±0.1	0.6±0.1	0.6±0.2
20:4ω6 (AA)	2.9±0.6^R^^P^	3.6±0.8^S^^P^	9.5±1.8^S^^R^	4.6±0.5	4.8±0.6	5.3±1.4	3.2±0.7^P^	3.3±0.6^P^	4.1±0.8^S^^R^
20:5ω3 (EPA)	5.4±0.6^P^	5.4±0.3^P^	3.8±0.7^S^^R^	5.4±0.6^P^	5.6±0.5^P^	7.4±1.3^S^^R^	3.7±0.9^P^	3.8±1.0	4.7±1.1^S^
22:4ω6	0.4±0.1^P^	0.4±0.1^P^	1.3±0.3^S^^R^	0.3±0.0^P^	0.4±0.1^P^	0.7±0.2^S^^R^	0.6±0.1^P^	0.6±0.1^P^	0.7±0.1^S^^R^
22:5ω3	1.2±0.1	1.1±0.1	1.2±0.2	0.9±0.1^P^	1.0±0.4^P^	2.4±0.9^S^^R^	1.3±0.3	1.2±0.4	1.4±0.2
22:5ω6	1.2±0.5^P^	1.6±0.4^P^	3.1±0.4^S^^R^	1.3±0.5^P^	1.2±0.5^P^	0.2±0.5^S^^R^	2.3±0.9	2.4±1.1	2.5±0.6
22:6ω3 (DHA)	25.4±2.1^P^	26.2±1.2^P^	18.8±4.8^S^^R^	28.1±2.2^R^^P^	25.3±3.3^S^^P^	22.8±1.6^S^^R^	32.7±7.4	33.5±8.6	32.4±5.9
Σ**PUFA**	**38.9±2.7**	**40.2±1.7**	**39.1±4.0**	**42.2±2.4**^R^	**40.0±2.5**^S^	**41.2±3.7**	**45.4±7.0**	**46.2±8.1**	**47.4±5.3**
Σω3	33.0±2.4^P^	33.5±1.5^P^	24.2±5.5^S^^R^	34.9±2.2^R^	32.5±2.8^S^	33.4±2.6	38.2±6.5	38.9±7.2	39.1±4.3
Σω6	5.9±0.8^R^^P^	6.7±1.2^S^^P^	14.9±2.1^S^^R^	7.3±0.8	7.5±1.0	7.7±1.5	7.1±1.4^P^	7.2±1.6	8.3±1.2^S^
Σω3/Σω6	5.7±0.8^P^	5.2±1.0^P^	1.7±0.7^S^^P^	4.8±0.6	4.4±0.8	4.5±0.8	5.5±1.2	5.6±1.4	4.7±0.4
DHA/EPA	4.8±0.5	4.8±0.3	4.9±0.9	5.3±0.8^R^^P^	4.5±0.8^S^^P^	3.2±0.5^S^^R^	9.8±3.9	9.9±4.7	7.4±2.9
DHA/AA	9.0±1.5^P^	7.6±1.7^P^	2.1±1.0^S^^R^	6.1±0.8^R^^P^	5.4±1.1^S^	4.5±1.0^S^	10.8±2.2^P^	10.3±2.2^P^	7.9±0.8^S^^R^
EPA/AA	1.9±0.2^R^^P^	1.6±0.3^S^^P^	0.4±0.2^S^^R^	1.2±0.2^P^	1.2±0.2^P^	1.4±0.3^S^^R^	1.3±0.5	1.2±0.6	1.2±0.5

Superscripts indicate significant differences among ovary phases for each tissue (*P* < 0.05 for ANOVA tests on arc-sinus root squared transformed data) with

^S^ different from SCP

^R^ different from RGN

^P^ different from OPS. Totals include minor fatty acid (<0.8%) components that are not shown.

ΣSFA from female albacore liver increased significantly during SCP and RGN (*F*_(2,43)_ = 7.67, *P <* 0.01) due to elevated 16:0. 18:0 was the second most abundant SFA but showed no significant variation among ovary phases. In contrast, ΣMUFA showed a decreasing trend from SCP to OPS (*F*_(2,43)_ = 8.07, *P <* 0.01), mainly due to significant decreased proportions of 18:1ω9, 18:1ω7 and 20:1ω9. While ΣPUFA did not exhibit any specific trend (*F*_(2,43)_ = 2.42, *P =* 0.10), DHA was significantly higher in the liver of SCP females and decreased until OPS. However, EPA was lower in SCP and RGN. This opposite trend in EPA and DHA levels led to lower DHA/EPA ratio in the liver of OPS females. A higher EPA/AA ratio was seen in the liver of RGN females mainly due to the increase proportion of EPA.

In albacore muscle, the proportion of ΣSFA was lower in SCP compared to OPS individuals (*F*_(2,41)_ = 5.18, *P <* 0.01) due to variations in 17:0 and 18:0. ΣMUFA did not show any significant variation with ovary phase (*F*_(2,41)_ = 0.90, *P =* 0.41) but rather exhibited high inter-individual variability. 18:1ω9 was the main MUFA and contributed to 7.8–11% of total fatty acids. ΣPUFA did not show significant variation with ovary phase (*F*_(2,41)_ = 0.17, *P =* 0.84) and the same was observed for DHA. However, compared to OPS females, Σω6 (including AA) and EPA were significantly lower in the muscle of SCP females. Thus, lower DHA/EPA ratio was also observed in muscle of OPS individuals.

### Differences in lipid and fatty acid composition between small- and large-sized ovary lobes

**Fig 3:**
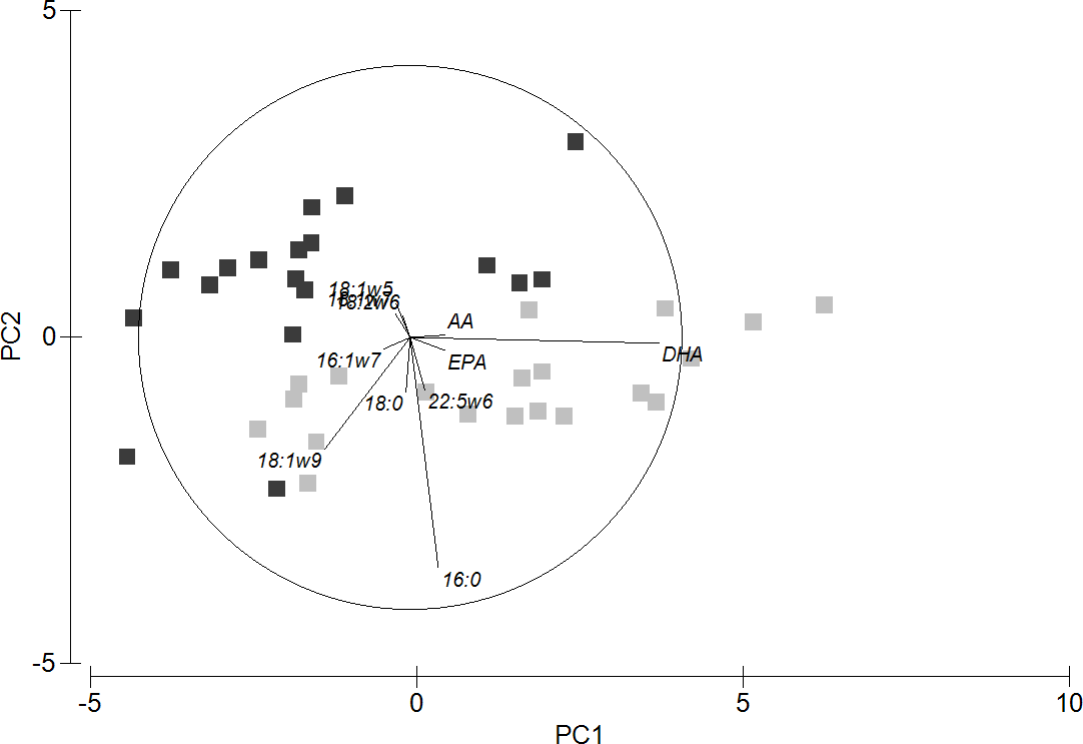
Scatterplot of principal component analysis for fatty acid data (expressed as % of total fatty acids) of large (dark grey) and small-sized (light grey) gonads of female albacore tuna caught in the waters of Mauritius. Principal Components (PC) 1 and 2 contributed to 81.5% of the variation.

**Fig 4:**
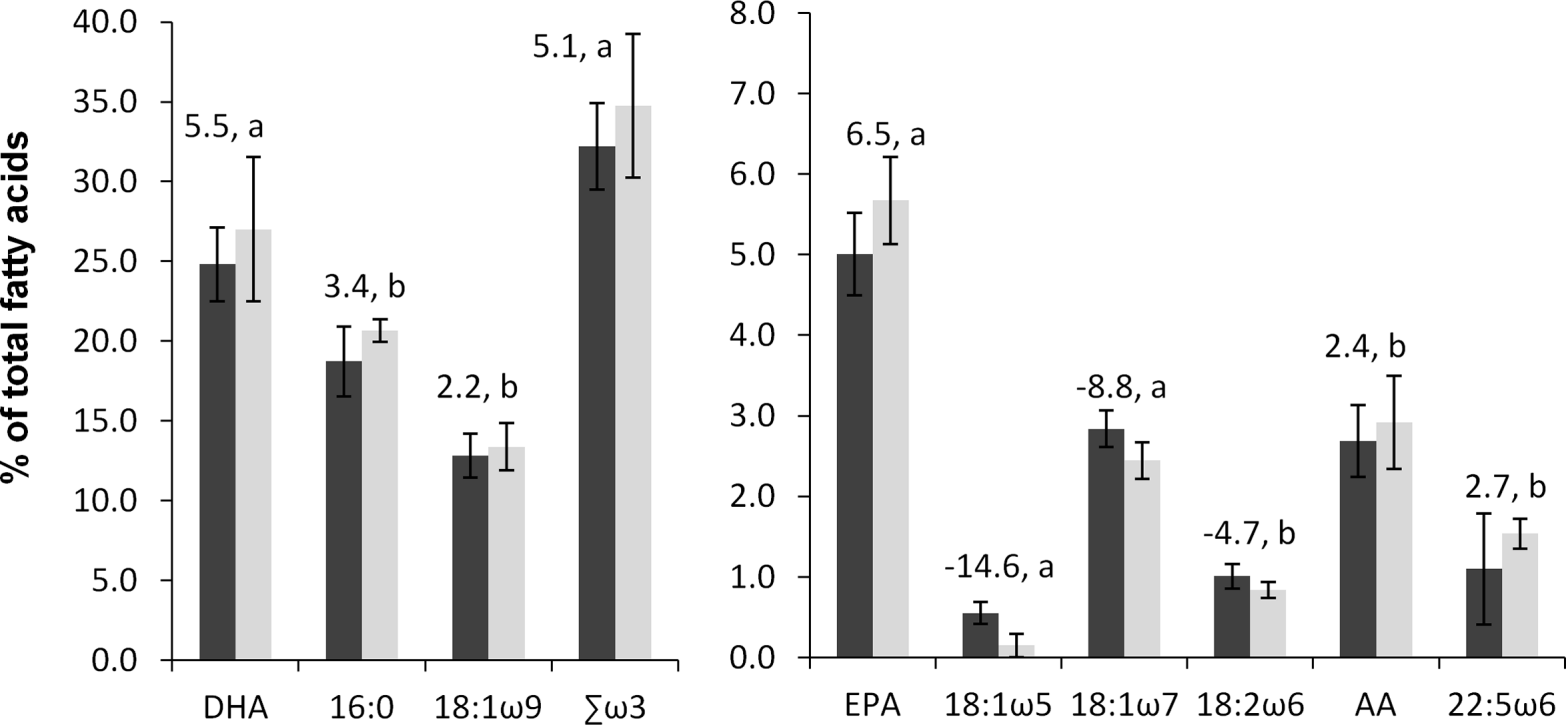
Mean % of the main fatty acids responsible for significant differences of fatty acid profiles between the large (dark grey) and small-sized (light grey) gonads of female albacore tuna caught in the waters of Mauritius. Values above the graph are the *t* values for paired t-tests and letters indicate *P* < 0.0001 (a) or *P* < 0.05 (b). Bars represent SD. *n* = 19.

The weight of the small and large ovary lobe pairs of albacore in the SCP were 137.7±38.3 g and 200.3±60.2 g (*n* = 29), respectively. PCA clearly showed two different groupings between the fatty acid profiles of small- and large-sized ovary lobes (Fig 3) which was confirmed by ANOSIM (Global-*R* = 0.361, *P =* 0.1%). SIMPER analysis revealed that the main fatty acid that led to the two groupings was primarily DHA which contributed to 61.5% to the observed dissimilarity. Other fatty acids including EPA, 16:0, 18:1ω9, 18:1ω5, 18:1ω7, 18:2ω6, AA and 22:5ω6 also contributed to the difference (see PCA and paired t-tests results in Figs 3 and 4, respectively). No significant difference in Σω6, TL (small ovary lobe: 44.9±7.0 μg mg^-1^; large ovary lobe: 43.9±9.6 μg mg^-1^, *n* = 20) and lipid classes was observed between pairs of ovary lobes, but Σω3 was significantly higher in the small-sized lobe (*t*_18_ = 5.1, *P* < 0.0001; Fig 4).

## Discussion

### Energy allocation strategy derived from fish condition and lipid dynamics

Our study shows that TL in female gonads of albacore were within the range observed in other tunas (0.8–6.5% *ww*; [3,23,42,43]). An accumulation of TL in the gonads of albacore during the SCP has been observed in other species showing asynchronous oocyte development such as in Atlantic bluefin tuna (*Thunnus thynnus thynnus*) [2], skipjack tuna (*Katsuwonus pelamis*) [23] and yellowfin tuna (*T*. *albacares*) [3]. This accumulation could in turn be due to a transfer of lipids from storage tissues to the ovaries via very low-density lipoproteins [4]. The mean TL of female albacore gonads in the SCP was however, nearly twice the amount of that reported for the tropical tunas, yellowfin and skipjack. Usually, species having an asynchronous oocyte development invest a small amount of energy into gametogenesis [44] and are thus expected to have low gonad TL. It is possible that the higher amount of energy seen in albacore reproduction may stem from the fact albacore has only one spawning season in austral summer [21,34], during which the energy acquired at specific feeding grounds, could be allocated entirely to reproduction. The tropical tunas, instead, have been described as having multiple spawning seasons throughout the year and females were required to feed intensively during the reproductive season to meet energy requirements [23,45]. The ovaries of SCP albacore, in particular, contain large proportions of WE, PL and TAG which started to accumulate in the lipid droplets during oocyte growth [1,4]. This prevalence of TAG and WE is a common occurrence in fish storing energy in the oil globules in their oocytes, such as in the case of albacore and other tuna species [3,23,46]. Similarly to these teleosts, PL also accumulated in the yolk of the oocytes in SCP albacore during vitellogenesis as it is an important requirement in the formation of cell and tissue membranes [1]. Thus, gonads of SCP female albacore, characterised by higher *I*_G_ [21], exhibit high TL, PL, WE and TAG, to ensure that sufficient endogenous resources are available for the metabolic activities in the developing embryo and larvae [46]. As the fish progresses to the regressing phase and finally to the regenerating phase, the amount of gonad TL, PL,WE and TAG gradually decreases as the advanced-stage oocytes are reabsorbed after spawning through atresia [21].

The liver plays an essential role in ovarian development in fish through the accumulation of a large amount of lipids, mainly in the form of TAG [47], derived from storage tissues or prey, during the process of vitellogenesis [2,4]. We observed that out of the spawning period, when albacore have the opportunity to accumulate energy derived from prey, the liver acted as a reserve for lipids, especially TAG and to a lesser extent, ST and PL. The lipids are stored in the fish hepatocytes as lipid droplets [48]. Low storage activity was however noted in the liver during SCP. It is likely that the accumulated TAG was mobilized from the liver to be allocated to the development of the ovary during the process of vitellogenesis and migratory nucleus as explained by the simultaneous raise in the gonads of SCP females, as described for yellowfin tuna [3]. With regards to ST and PL, a similar process could also occur but the allocation of these lipid classes to the gonads could have been more consequent in earlier oocyte maturity stages, at pre-vitellogenic or early vitellogenic, where structural compounds were required for oocyte formation and lipoprotein synthesis [49]. The same trend for liver PL was observed in the Atlantic bluefin [2] and yellowfin tunas [3]. In contrast to the tropical tunas, we found no significant variations in liver WE with ovary phase. The high levels of WE found in the SCP ovaries could have been derived from long-term storage tissues of albacore where energy is stored mostly in the form of WE [2]. In albacore, we found a decreasing trend of TL in the liver with *I*_G_. An opposite relationship was observed in skipjack tuna, considered as an income breeder and where ovary growth is not dependent on hepatic energy reserves but rather on food intake [23]. Albacore, on the other hand, appeared to rely mostly on lipid reserves from the liver in our study, which is a characteristic of capital breeders [50]. A slight increase in liver weight with gonad maturation, reflected in but positive trends between *I*_H_ and *I*_G_, gonad TL, PL and ST, was also observed in the present study. This is in turn related to the relative increase in liver weight as a result of stimulation of lipoprotein biosynthesis by hormones during gonad maturation [17] and is related to the role of liver as a reservoir of lipids. The role of liver as a lipid depot has also been demonstrated in pouting, *Trisopterus luscus* [51], described as a capital breeder. Fishes use the liver as the main site for synthesis of egg constituents which are then carried to the gonad for uptake [52]. Vitellogenin, which is the egg yolk precursor, has the ability to transport lipid as lipoprotein throughout the body of eukaryotic organisms. The importance of *I*_H_ as an indicator of reproductive success in Atlantic cod (*Gadus morhua*) was pointed out by Marshall et al. [10]. Consequently, these studies support the idea that the liver represents one of the main source of energy for albacore. The liver also contained a greater proportion of FFA compared to the other tissues, as a result of high lipid catabolism. These FFA may be used to provide energy or reacylated into lipid classes [53].

Mean TL in white muscle of female albacore was relatively low in our study (<1% *ww*), as seen in other tunas [3,23,54], but lower than that reported in albacore sampled from the Pacific Ocean (1.2–2.6% *ww*; [27]) and from Reunion Island in the Indian Ocean (0.8–6.4% *ww*; [55]). The TL was even lower compared to smaller albacore from the Pacific Ocean (average fish weight: 6.07 kg; 0.67–18.74% *ww*; [56]) and from the South African coast (average fish weight: 11.0 kg; 3.7–13.5% *ww*; [55]). High amounts of lipids in the somatic tissue is a typical characteristic of capital breeder species from temperate regions, such as the bluefin tuna [2], where the lipid deposits may be used later for metabolic activity during migration or for reproduction. Although white muscle TL from western Indian Ocean albacore varied to a large extent among individuals, regardless the ovary phase, the highest observed maximum values reached 2.3% *ww* only. Moreover, even if none of the lipid classes in the muscle showed significant differences with ovary group, their variation along the ovary phases was more apparent with *I*_G_ of the individual fish revealing increased TL and TAG with increasing *I*_G_. An increase in the concentration of TAG has also been observed in the white muscle of bluefin tuna with gonad development and was attributed to their high aerobic capacity [2]. Lipid reserves in muscle of SCP females could indicate that these fish had better condition which may heighten their fecundity as proposed by Schaeffer [57]. As condition is linked to fecundity [58], the positive correlations of gonad TL and TAG with *F*_K_ and the associated negative correlations of liver TL, PL and TAG with *F*_K_ suggest that albacore in better condition could contribute more into reproduction.

From our study, mature albacore tuna displayed a low energy storage in the muscle tissues. Furthermore, during sampling activities, we observed that albacore did not appear to change its feeding habits even during the SCP and continued foraging. Intensive feeding throughout ovary maturation allows the incorporation of dietary lipids for development of the oocytes [59]. These evidences would normally suggest that albacore could be considered as an income breeder. However, results from other studies as discussed above, revealed the ability of immature albacore to store large amount of lipids in their muscle tissues, and our study also demonstrated the high storage capacity of its liver. It was even suggested that the liver acts as a buffer to ensure maturation of ovaries at low rates of food (energy) intake [60]. Pure income breeders such as skipjack tuna [23], *Pagrus pagrus* [44] and *Merluccius merluccius* [61], are species that exhibit a higher dependence on food during spawning for successful reproduction. Both bluefin [2] and albacore [30] tunas have previously been described to have a large amount of energy reserves in the form of perigonadal fat. Ratty et al. [30] found that in some albacore individuals, the perigonadal fat was rudimentary while in others, it was conspicuous and well-developed being white and creamy, and could even extend beyond the gonad. The fat body was observed to be proportionate to fish size, being larger in the immature (meiotically inactive) compared to those that were meiotically active. Their results reflect the use of lipids from the perigonadal fat for gonad development such as in bluefin tuna where lipids are transferred to the gonad and catabolized to provide energy [2]. Since our study did not include immature albacore, it is not possible for us to assess the perigonadal and muscle fat content to confirm this transfer of energy to the gonads during the pre-spawning period. The fact that we did not find perigonadal fat in any of the females caught in the waters of Mauritius, confirms the depletion of energy from perigonadal fat depots once the fish has attained maturity and is in the SCP. Likewise, in the South Pacific albacore, a decrease in energy available for reproduction can be observed along the spawning season through a reduction in the batch fecundity, with higher batches occurring at the start than at the end of the spawning season [34]. Previous investigations have also demonstrated a depletion in the fat reserves of albacore after spawning [62,63] supporting the fact that the energy to be allocated to reproduction is stored well before and will be mobilized at a later time to support the energy-demanding processes associated to reproduction. In the same instance, other studies of other species found increased energy reserves immediately before gonad ripening which were then depleted at the end of the spawning season [51,64,65]. Interestingly, perigonadal fat was also absent in OPS female albacore from the present study. These OPS were caught in May and June shortly after the spawning season (October to January) [21] and it is thus probable that energy stores, including the perigonadal fat, are fully replenished once on specific feeding grounds as proposed by [21] such as in the regions located north of 10°S, where nutrient-rich waters occur as a result of upwelling [66]. This is highly probable since studies on albacore from other regions have shown that they do undertake extensive feeding migration [62,63].

Breeding strategies are usually not restricted to extreme forms of capital and income breeders but rather an array of intermediate strategies [14]. Between these two extremes are the capital-income breeders which are characterized by large energy stores but which exhibit important feeding activity during spawning. Eventually, our results clearly show that the liver of albacore acts as an energy storage for future ovarian development. However, to partially offset the cost of reproduction during the spawning season, additional energy appears to be required from concurrent feeding. Storing energy in the form of fat can provide organisms with the added advantage to adapt to changes in their environment. Albacore which is a temperate tuna spending a large proportion of its life cycle in the tropical and sub-tropical waters [19], may have resulted to this species in developing a combined strategy rather than being strictly capital like the temperate bluefin tuna. It appears to use a strategy that more closely aligns to capital breeding than for instance, yellowfin tuna which was suggested to be an income-capital breeder [3]. Thus, albacore can be viewed as a capital-income breeder. Other temperate species have also been proposed to use a capital-income strategy such as sole (*Solea solea*) and the Northern anchovy (*Engraulis mordax*) [14].

### Albacore fatty acid requirements for spawning

Spawning and egg quality are determined by lipids which provide essential fatty acids needed for growth and survival [8]. The fatty acid profiles of albacore gonads and liver in this study are similar to that reported for bluefin tuna in the spawning season [2]. However, since the latter study did not include regenerating females, it was not possible to make interspecific comparisons between SCP and OPS females. The profile of fatty acids in somatic and reproductive tissues of female albacore from western Indian Ocean was characterised by the predominance of DHA, 16:0, 18:0 and 18:1ω9, but low proportions of ω3 highly unsaturated fatty acids. All these fatty acids, with the exception of DHA which is found in polar lipids [67], are generally found in the neutral lipids of many other marine fish [53] which preferentially accumulate high levels of SFAs and MUFAs in their lipid reserves [68], that is in the oil droplets of their oocytes [4] in the case of some teleosts such as albacore. Since TAG and WE were highest in SCP gonads of albacore, it is not surprising that the levels of these fatty acids, especially 18:1ω9, showed the same trend.

The liver of albacore was richer in 16:0 during the SCP phase. SFAs and MUFAs, especially 16:0 [46], are usually the preferred substrates during β-oxidation in the liver, which is the centre of metabolic reactions, to provide energy in fish [64], and are often accumulated in fish gonads [4]. In addition to their role as an energy source, MUFAs play a suite of physiological roles such as hormone mobilisation, maintenance of buoyancy and permeability control [69]. Compared to liver tissue, no significant difference in the proportion of 16:0 was identified in both gonads and muscle, but the proportion of 18:0 was greater in the gonads of OPS females. In female Atlantic bluefin tuna [2], an accumulative pattern of both 16:0 and 18:0 was observed in the gonads during the ovary phases with peaks just prior to SCP. It is known that some specific fatty acids are preferentially conserved, converted and used in fish gonads [70]. It is likely that in female albacore, 16:0 is being conserved in the oocytes mainly as endogenous reserves for the developing embryo and larvae while the 18:0 is being used as an energy source for the development of the vitellogenic oocytes. Large amounts of 18:1ω9 could also be found in albacore tissues and is thought to be produced during *de novo* synthesis of fatty acids in fish [71]. Its low proportion in the liver is consistent with its accumulation in the gonads during the SCP. MUFAs generally do not have a structural role, but are preferentially used for oxidation to release energy [4]. Ortega and Mourente [46] showed that MUFAs, especially 16:1ω7 and 18:1ω9, decreased to a large extent during larval development in Atlantic bonito, *Sarda sarda*, confirming their importance as a source of energy after hatching.

From our results, it is clear that albacore, like other tunas [2,72,73], accumulate DHA which is preferentially conserved in all tissues. This accumulation of DHA, derived from their prey, may be considered as a characteristic of tunas due to their position of top predators in the marine food chain [74,75]. It is widely accepted that fish roe contain copious amounts of long-chain PUFAs of the ω3 type [1,53] as seen in albacore. These ω3 PUFAs act as structural elements in biomembrane formation in developing oocytes [2,17]. Compared to OPS individuals, both DHA and EPA were significantly higher in the gonads of SCP females which were characterised by a DHA/EPA ratio of 4.8/1. This ratio is higher compared to those found in fertilised eggs of the Atlantic bonito (2.7/1; [46]) but similar to the gonads of the Atlantic bluefin tuna during spawning (4.5/1; [2]). Essential fatty acids play an important role in the maintenance of cellular structure and function in fish [7,17,53] and DHA in the development of brain tissue and the retina [76,77]. A low DHA/EPA ratio can lead to an imbalance in the membrane phospholipids which may in turn affect larval growth and quality. This is because EPA and DHA are thought to compete for the same enzymes involved in the esterification of fatty acids into phospholipids in cell membranes [76,78].

Typically AA is concentrated into fish oocytes to help meet the demands of the developing embryo and larvae [53,79]. Contrarily to EPA and DHA, lower proportions of AA were observed in SCP gonads of albacore leading to a lower proportion of AA in relation to EPA (i.e. EPA/AA of 1.9/1) compared to OPS females (0.4/1). Higher ratios of EPA/AA were found in the SCP gonads (containing hydrated oocytes) of the Atlantic bluefin tuna (4.9/1) and fertilised eggs of Atlantic bonito (7.7/1) [46]. The variation of AA along the ovary phases of albacore is similar to that of Atlantic bluefin tuna as studied by [2] with lower proportions of AA being recorded in SCP ovaries containing late vitellogenic or migratory nucleus oocytes. Mourente et al. [2] however showed significantly higher proportions of AA in the ovary of bluefin tuna containing oocytes in the pre-vitellogenic stage. The decline in the relative concentrations of AA to EPA in albacore gonads during the SCP may result from interactions between ω6 and ω3 fatty acids where they exhibit an inhibitory bioconversion effect on each other [6]. After the spawning season, when EPA is not relocated from the somatic tissues to be concentrated into the gonads and its proportion falls in the gonads, the proportion of AA may rise again as it is preferentially integrated into phospholipids [77] leading to lower EPA/AA ratio. The opposite trend then occurs in the liver with increased proportion of EPA after the spawning period, accompanied by a higher EPA/AA ratio in OPS females.

The small-sized ovary lobe was found to have slightly higher TL, and significantly higher proportions of essential and key fatty acids (DHA, EPA and AA, as well 16:0 and 18:1n9) than the large-sized one. Such a finding suggests that the small-sized lobe could contribute more to the reproductive success, a characteristic that may result from a similar strategy in male albacore where higher meiotic activity in the smaller testis lobe has been reported [30]. Ratty et al. [30] were among the first to have described the aspect of gonad dimorphism in albacore and they found that the right gonad lobes were usually larger in cross-section and volume than those on the left while the length of both gonad lobes remained the same. They also found that the size of both left and right testes increased exponentially with *L*_F_ and had similar rates of increase in area. In addition, in fish where perigonadal fat was observed, the associated fat was larger for the larger ovaries. In an energetic aspect, this difference in amount of fat may be attributed to the increased activity of the smaller ovary lobe which requires more energy than the other less active lobe. Usually, the size of the gonad lobes in other tunas is nearly symmetrical. To our knowledge, no information is available in the literature to explain the advantage of fish to have a more active gonad lobe during reproduction. Ratty et al. [30] proposed that the smaller gonad lobe could be kept in an advanced reproductive state for spawning “opportunistically” while the large lobe matures only when the fish is at appropriate spawning grounds. They hypothesized that this would allow albacore to extend their reproductive potential with low energy expenditure. However, no study found differences in maturity stage between ovary lobes and gonad dimorphism was still observed even on their specific spawning grounds (e.g., [21,32]). Recent biological studies have appraised gonad dimorphism in albacore and conducted fecundity estimates for both the small- (left) and large-sized (right) gonads to obtain more accurate individual batch fecundity (e.g. [32,34]). Indeed, differences in the count of migratory nucleus or hydrated oocytes between gonad lobes were found [32]. Several studies also reported gonad asymmetry in other teleosts at the time of the breeding season [80,81].

Our study is the first one to investigate gonad dimorphism in fish based on TL and its constituent fatty acids while others have described mainly the morphological aspects [22,82,83]. Notably, the study shows that lipid and fatty acid analysis can be used as a means to provide detailed information on morphological observed differences.

## Conclusion

Our results provide evidence that lipids and their constituent fatty acids in the reproductive and somatic tissues of mature albacore vary along the ovarian phases reflecting lipid utilization during gonad development. This process is in turn linked to the reproductive strategy of albacore which uses a capital-income breeder, relying mostly on stored energy for reproduction and on supplementary energy derived from feeding for the later gonadal development stages or other batches along the spawning season to achieve successful reproduction. The extent and influence of the recently assimilated energy to reproduction still needs to be determined and the type of energy available (from storage tissues or recent assimilation) for allocation to reproduction or maintenance remains unknown. Further energetics studies that include immature albacore and lipid profiling of their perigonadal fat, as well as post-spawning albacore from possible feeding grounds in the region, could provide more information on the energy allocation strategy of albacore. Moreover, the difference in the amount of lipids catabolized may also vary with the presence of other energy sources. Additional biochemical studies on the protein and carbohydrate contents in female albacore may provide more information on their energy dynamics during the reproductive cycle. The difference noted between the small- and large-sized ovary lobes of albacore should be taken into consideration in future reproductive and biochemical studies. Studies focusing on the benefits of gonad dimorphism in fish are important and may provide further insights into the energy allocation during reproduction. Future work should seek to understand how projected increases in seawater temperature and associated changes in phytoplankton community, composition, abundance, may affect the availability of fatty acids to albacore that could in turn influence the reproductive potential and thus productivity of local stocks.
